# Comprehensive characterization of DNA methylation changes in Fuchs endothelial corneal dystrophy

**DOI:** 10.1371/journal.pone.0175112

**Published:** 2017-04-06

**Authors:** Emily Khuc, Russell Bainer, Marie Wolf, Selene M. Clay, Daniel J. Weisenberger, Jacquelyn Kemmer, Valerie M. Weaver, David G. Hwang, Matilda F. Chan

**Affiliations:** 1 Department of Ophthalmology, University of California, San Francisco, California, United States of America; 2 Department of Surgery and Center for Bioengineering and Tissue Regeneration, University of California, San Francisco, California, United States of America; 3 Bay Area Physical Sciences-Oncology Program, University of California, Berkeley, California, United States of America; 4 Department of Biochemistry and Molecular Medicine, University of Southern California, Los Angeles, California, United States of America; 5 Helen Diller Family Comprehensive Cancer Center, University of California, San Francisco, California, United States of America; 6 Departments of Anatomy and Bioengineering and Therapeutic Sciences and Eli and Edythe Broad Center for Regeneration Medicine and Stem Cell Research, University of California, San Francisco, California, United States of America; 7 Francis I. Proctor Foundation, University of California, San Francisco, California, United States of America; Wayne State University, UNITED STATES

## Abstract

Transparency of the human cornea is necessary for vision. Fuchs Endothelial Corneal Dystrophy (FECD) is a bilateral, heritable degeneration of the corneal endothelium, and a leading indication for corneal transplantation in developed countries. While the early onset, and rarer, form of FECD has been linked to *COL8A2* mutations, the more common, late onset form of FECD has genetic mutations linked to only a minority of cases. Epigenetic modifications that occur in FECD are unknown. Here, we report on and compare the DNA methylation landscape of normal human corneal endothelial (CE) tissue and CE from FECD patients using the Illumina Infinium HumanMethylation450 (HM450) DNA methylation array. We show that DNA methylation profiles are distinct between control and FECD samples. Differentially methylated probes (10,961) were identified in the FECD samples compared with the control samples, with the majority of probes being hypermethylated in the FECD samples. Genes containing differentially methylated sites were disproportionately annotated to ontological categories involving cytoskeletal organization, ion transport, hematopoetic cell differentiation, and cellular metabolism. Our results suggest that altered DNA methylation patterns may contribute to loss of corneal transparency in FECD through a global accumulation of sporadic DNA methylation changes in genes critical to basic CE biological processes.

## Introduction

The cornea serves as the main refractive element of the mammalian visual system by focusing incoming light through the lens onto the retina. The cornea is composed of three major layers: epithelium, stroma, and endothelium [1]. The corneal endothelium is the innermost layer of the cornea and consists of a monolayer of polygonal endothelial cells attached to a basement Descemet’s membrane composed primarily of Type IV collagen [1]. The corneal endothelium functions to maintain corneal clarity by regulating corneal hydration status through a pump-leak mechanism [2]. Ion pumps expressed in corneal endothelial cells actively transport fluid out of the cornea to preserve stromal dehydration and corneal transparency [2]. Cell adhesion molecules contribute to the electrical coupling of corneal endothelial cells (CECs), and also enable the CECs to function as a barrier to fluid movement into the cornea [3]. CECs contain large numbers of mitochondria and are the most metabolically active cells in the cornea [4]. Because CECs have limited proliferative and regenerative capacity [3], damage to these cells results in a reduced capacity to pump fluid out of the corneal stroma, loss of corneal clarity, and decreased visual acuity [4]. The only definitive treatment option to restore vision following CEC damage is corneal transplantation with surgical procedures including Descemet’s Stripping Automated Endothelial Keratoplasty (DSAEK) and Descemet’s Membrane Endothelial Keratoplasty (DMEK) [5, 6].

Fuchs Endothelial Corneal Dystrophy (FECD) is a bilateral, heritable degeneration of the corneal endothelium characterized by progressive development of focal excrescences of Descemet’s membrane termed “guttata”, endothelial cell dropout, progressive corneal edema, and loss of vision [7, 8]. Endothelial dystrophies are the most prevalent type of corneal dystrophy, and FECD is the most common of these endothelial dystrophies and is one of the major indications for corneal transplant surgery in the United States (US) and other Western Countries [9–12]. This disease can first appear in patients around 30 to 40 years of age, with vision starting to become affected by the disease around 50–60 years of age. The prevalence of FECD has been estimated at about 5% among persons over the age of 40 years in the US [13]. In the US, FECD is the leading indication for corneal transplantation in the geriatric population [14].

FECD is a complex disease whose pathogenesis is due to genetic and environmental factors [15]. The early onset form of FECD is the rarer form of disease and has been linked to *COL8A2* mutations [16]. The classic, late onset form of FECD is the more common form and is a genetically heterogeneous disease. Although the genes *SLC4A11 [16], TCF8 [17], LOXHD1 [18],* and *AGBL1* [19] have been linked to late onset FECD, they are responsible for only a minority of cases. Traditional linkage studies and genome-wide association studies have recently identified an expanded trinucleotide repeat in the third intron of transcription factor 4 (*TCF4*) to also be strongly associated with late onset FECD [20–23].

Epigenetic modifications that occur in FECD are unknown. Epigenetic modifications can drive heritable changes in gene expression that are not accompanied by changes in DNA sequence [24]. DNA methylation and chromatin remodeling are examples of epigenetic modifications and have been shown to be important in normal ocular processes including the development of the retina and lens [25]. Aberrant DNA methylation changes have been associated with ocular diseases including the development of pterygia [26], macular degeneration [27], retinoblastoma [28], and uveal melanoma [29]. DNA methyltransferases (DNMTs) are a family of enzymes that catalyze the transfer of a methyl group to DNA and all three family members are highly expressed in the cornea suggesting the importance of DNA methylation in normal corneal function [30].

In the present study, we tested the hypothesis that changes in DNA methylation consistently occur within the endothelial tissue of patients with late onset FECD. We uncovered DNA methylation changes in FECD that affect specific genes and gene families that are crucial to normal barrier and fluid transport functions of the CE.

## Results

### Comparison of genome-scale DNA methylation profiles in the corneal endothelium of FECD patients and controls

The endothelial tissue of 15 patients with FECD undergoing corneal transplantation by endothelial keratoplasty (13 DSAEK and 2 DMEK) was collected at the time of surgery and promptly processed for DNA isolation and bisulfite conversion. Following processing, 9 of the 15 endothelial samples (60%, 8 DSAEK and 1 DMEK) had sufficient DNA extracted for array analysis due to the low number of healthy endothelial cells associated with FECD. Four age- and gender-matched, normal endothelial tissue samples were processed simultaneously as controls. The clinical findings of the FECD and control patients are summarized in Table 1. The nine FECD samples analyzed were from five male and four female FECD patients, and the controls were obtained from two males and two females. The average age of the FECD and control patients was 64 years and 71 years, respectively. The age range for the FECD patients was 47.7–83.9 years and for the controls was 46.2–75 years. The FECD patients had an average guttata grade of 2.6 and corneal thickness of 650 microns, while the control samples did not have guttata.

**Table 1:** Demographics of study participants for genome-wide DNA methylation analysis.

	All FECD (n = 15)	Control (n = 4)	Analyzed FECD (n = 9)
**Age (SD)**	67.73 (10.24)	70.56 (9.96)	64 (10.98)
Age range	47.7–83.9	46.2–75	47.7–83.9
**Sex**			
Male	7 (47%)	2 (50%)	5 (56%)
Female	8 (53%)	2 (50%)	4 (44%)
**Study Eye**			
Right	8 (53%)	2 (50%)	3 (33%)
Left	7 (47%)	2 (50%)	6 (67%)
**Procedure**			
DSAEK	13 (87%)	0 (0%)	8 (89%)
DMEK	2 (13%)	4 (100%)	1 (11%)
**FECD Measures**			
Guttae	2.6	0	2.56
Pachymetry	650	N/A	628.38

**Fig 1:**
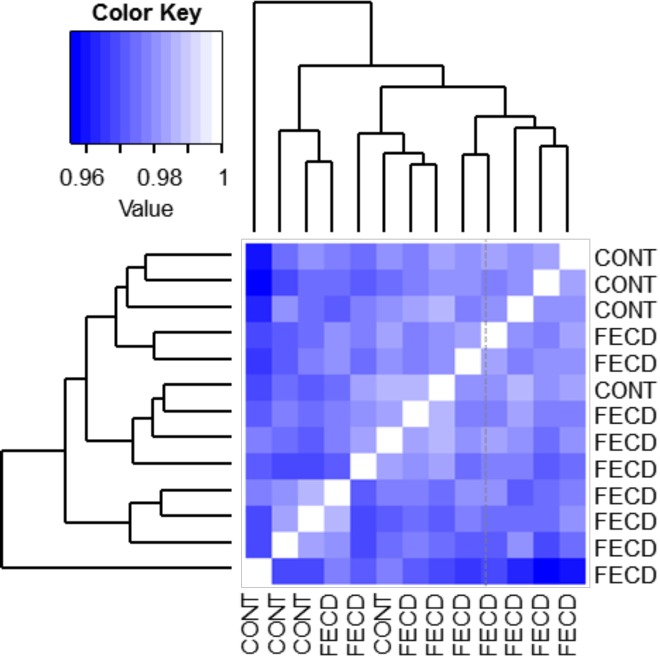
Heatmap visualization of pairwise Spearman correlation coefficients comparing FECD and control corneal endothelium samples with dendrogram to show clustering (Euclidean distance). Control and FECD samples separate according to disease variable, with the exception of one FECD sample.

We performed genome-scale DNA methylation analysis using the Illumina Infinium Human Methylation450 Array (HM450) on the bisulfite-treated DNA samples to profile the DNA methylation status of over 482,421 probed sites spanning 99% of RefSeq genes. Sample pairwise correlation and hierarchical clustering revealed that genome-wide DNA methylation patterns in FECD are distinct from those observed in normal corneal endothelial tissue (Fig 1). To determine if age, sex, pachymetry, or guttata grading had a significant effect on DNA methylation status, we next performed a nonparametric principal component analysis to determine if variance could be attributed to these clinical variables. The first four principal components capture 98.4% of the variance observed between samples, but none of these components was clearly associated with known clinical variables within the cohort (data not shown). These data indicated that while DNA methylation profiles are distinct between FECD samples and controls, this is not likely to be due to the clinical status of age, sex, pachymetry, or guttata grading.

### DNA methylation profiles of individual genes

**Fig 2:**
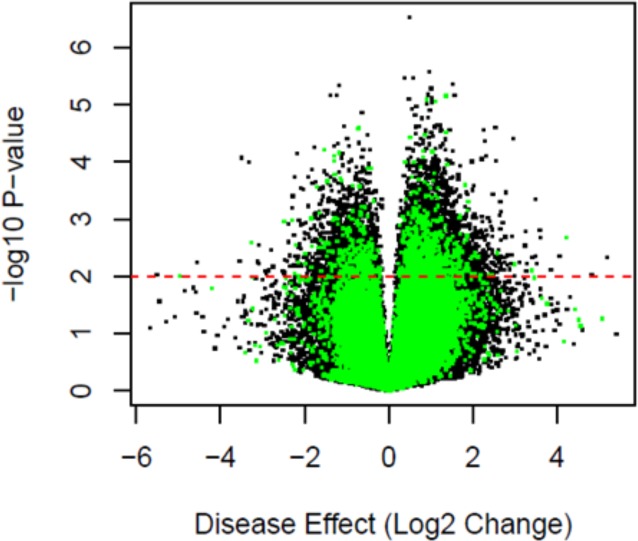
Volcano plot showing statistical significance and fold change effect of individual FECD probes compared to control. The red line indicates threshold of significance where Q ≤ 0.01.

We next compared the DNA methylation levels of individual probes between FECD cases and controls. Previous studies have identified genes and chromosomal regions linked to late-onset FECD, so we queried our data for the DNA methylation status of *COL8A1*, *TCF4*, *SLC4A11*, and *AGBL* genes [31]. We did not find a statistically significant difference in the DNA methylation levels for the majority of the probes annotated to genes previously associated with FECD (data not shown). Among the four genes, *SLC4A11* had the highest proportion of significantly differentially methylated probes (14 out of 36 probes). Significant DNA methylation differences were identified for numerous other probes (10,961 probes, FDR = 0.01; Fig 2). Outlier probes representing individual extreme DNA methylation changes between the two groups were not identified (Fig 2). Instead, numerous loci across the genome had a difference in DNA methylation status (FDR = 0.01).

**Fig 3:**
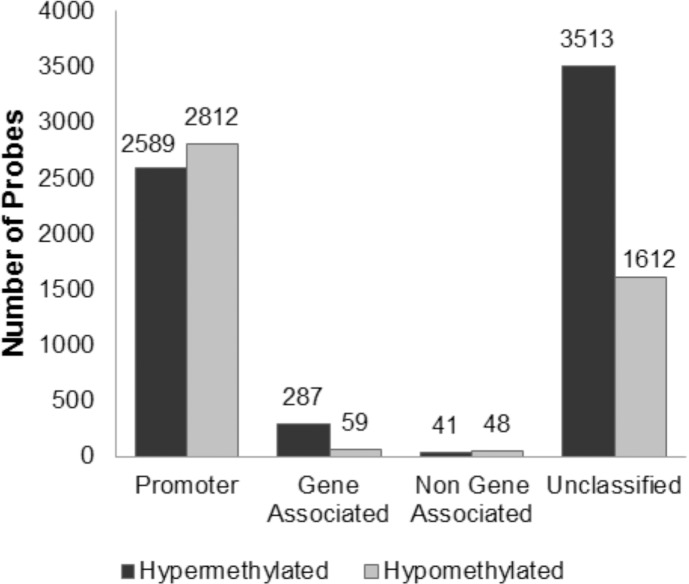
Number of differentially methylated probes between FECD and control samples (P < 0.05), grouped by the targeted genomic regions.

We identified a total of 10,961 differentially methylated probes in the FECD samples compared with the control samples (FDR = 0.01, Fig 3). A majority of these probes (6,430; 59%) were hypermethylated in the FECD samples, while 4,531 (41%) were hypomethylated in the FECD samples (Fig 3). Of these, 5,747 probes were located in either gene promoters or gene bodies, while 89 probes were located in intronic gene regions (Fig 3). Notably, most of the differential DNA methylation occurred at probes that are annotated to intergenic regions (Fig 3). Furthermore, no probes were significantly differentially methylated with respect to age or sex in the framework of the linear model (FDR = 0.1), further suggesting that these are not major drivers of DNA methylation differences observed in FECD patients.

Table 2 lists the 20 most highly differentially methylated probes that target protein-coding genes. Of these, nine target genes have roles in cytoskeletal organization including *MTUS2*, *COBL*, *CDH4*, *BSN*, *CCDC124*, *EML3*, *KIF26A*, *ZNF135*, and *NEGR1*. Six of the 20 genes have roles in signal transduction and cellular metabolism including *PDE11A*, *GNAS*, *GUCY2C*, *FSAN*, *GAA*, and *SYT16*.

**Table 2:** Most highly differentially methylated probe sites.

Gene	Probe ID	Function	FECD Coeff	P Value^a^	Q Value
PDE11A	cg02819655	Phosphodiesterase	-5.4544	*	0.4959
CCDC57	cg25388952	Coiled-coil Domain	5.1876	**	0.4613
GNAS	cg09885502	G Protein	-4.8458	*	0.4811
MTUS2	cg13506281		4.8262	**	0.469
		Microtubule Associated			
C1orf94	cg00124902	Open Reading Frame	-4.6493	*	0.4775
COBL	cg25543264	Actin Cytoskeleton Organization	-4.5819	*	0.4816
SPG21	cg25879395	CD4 Receptor Binding	-4.5692	*	0.5179
NME6	cg08146865	NDP Kinase	-4.5483	**	0.4632
CDH4	cg00704664	Cadherin	4.3053	*	0.4902
MYADML	cg04131969	Pseudogene	4.2389	*	0.5105
GUCY2C	cg00267207	Enterotoxin Receptor	4.2207	**	0.4582
BSN	cg05126514	Presysnaptic Scaffold	-4.2144	*	0.4782
CCDC124	cg14060113	Cytokinetic Organization	4.0241	*	0.4989
EML3	cg11755407	Microtubule Dynamics	4.0021	*	0.5116
KIF26A	cg09856996	Microtubule Binding	3.894	*	0.5145
ZNF135	cg23499373	Cytoskeletal Organization	3.8765	*	0.5007
FASN	cg03407524	Fatty Acid Catalyst	3.8595	**	0.4653
GAA	cg16464924	Glycogen to Glucose Enzyme	3.7704	*	0.5003
NEGR1	cg09664314	Cell-Adhesion	3.708	*	0.5221
SYT16	cg05859760	Secretory Vesicle Trafficking	3.633	*	0.4918

^a^* P ≤ 0.05

** P ≤ 0.01

*** P ≤ 0.001

### Ontological enrichment amongst differentially methylated genes

In order to better understand biological changes that occur in FECD, we next assessed whether differentially methylated genes were disproportionately involved in specific ontological categories. The Open Biomedical Ontologies public database was used to categorize the genes with DNA methylation probe sites into larger related gene families based on biological processes, while still separating probes by location within the gene. GOseq [32, 33] was used to identify biological processes disproportionately represented among the genes and promoters containing sites with significant DNA methylation changes in FECD. DNA methylation changes can either positively or negatively affect gene expression depending on its gene location. Promoter DNA methylation may result in gene silencing while gene body methylation is positively correlated with gene expression [34]. In promoter regions, gene ontology sets were enriched in DNA hypomethylation changes in FECD CE. The opposite was true for the gene bodies, where FECD CE showed DNA hypermethylation changes (data not shown).

**Fig 4:**
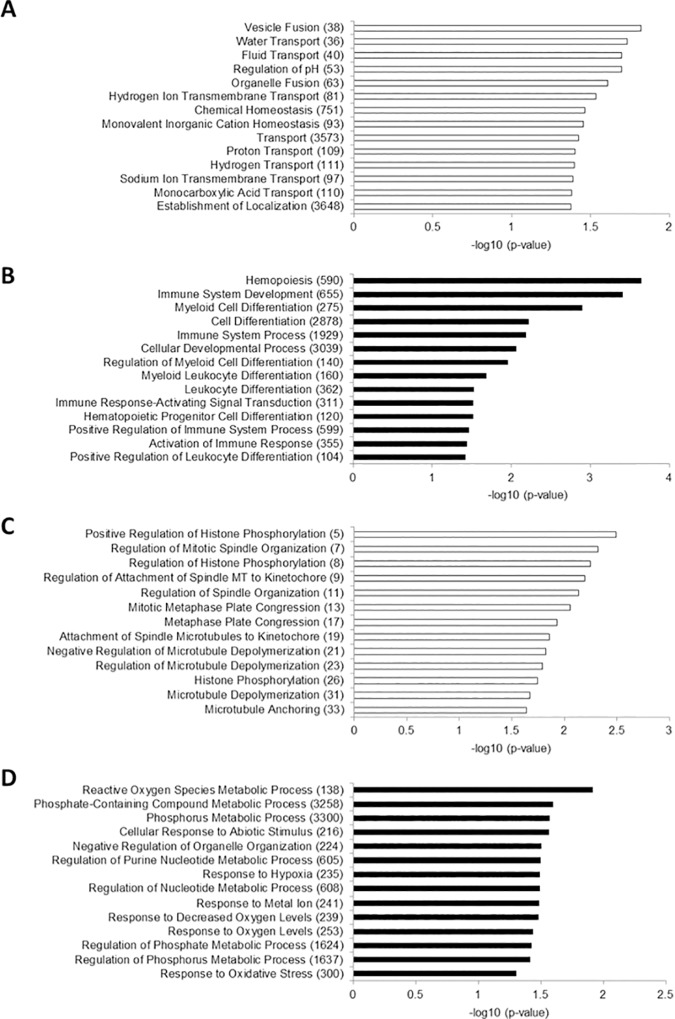
Gene Ontology (GO) categories most strongly enriched among probes in FECD CE. Number of represented probes in each category is in parentheses after category name. (A) Gene body DNA hypomethylation, (B) Gene body DNA hypermethylation, (C) Promoter DNA hypomethylation, and (D) Promoter DNA hypermethylation.

In general, DNA methylation changes did not closely correlate with individual pathways. The most strongly enriched ontologies among differentially methylated promoter and gene body regions were categories containing few genes assayed by a small number of probes, suggesting a high false positive rate and that no pathways show consistent DNA methylation in FECD. However, among the subset of enriched categories that contained large numbers of genes, many correspond to biological functions necessary for the unique functions of the corneal endothelium. Specifically, gene body DNA hypomethylation was disproportionately present in genes involved in fluid and ion transport in the FECD samples, the largest category being Transport (p = 0.0376) (Fig 4A). This result is consistent with prior studies that have found reduced expression of gene families associated with ion and water transport in the CE of FECD patients including bicarbonate transporter-related protein-1 (*BTR1*) [35], aquaporin-1 (*AQP1*) [36], and monocarboxylate transporters (MCTs) [37]. By contrast, gene body DNA hypermethylation was associated with hematopoetic cell differentiation in the FECD samples, especially related to Immune System Processes (p = 0.0065) (Fig 4B). Promoter DNA hypomethylation in the FECD samples also showed enrichment of gene families related to cytoskeletal organization, such as Microtubule Anchoring (p = 0.0229) and Microtubule Depolymerization (p = 0.0213) (Fig 4C). Proper expression of cytoskeletal factors is necessary for the function of CE to restrict fluid leakage into the corneal stroma [3]. Promoter DNA hypermethylation was observed in gene families involved in metabolic processes, including Reactive Oxygen Species Metabolic Processes (p = 0.012) and Phosphorus Metabolic Processes (p = 0.027) (Fig 4D). This result correlates with the prior finding that mitochondrial transcripts are depleted in FECD [35].

### Validation with MethyLight

**Fig 5:**
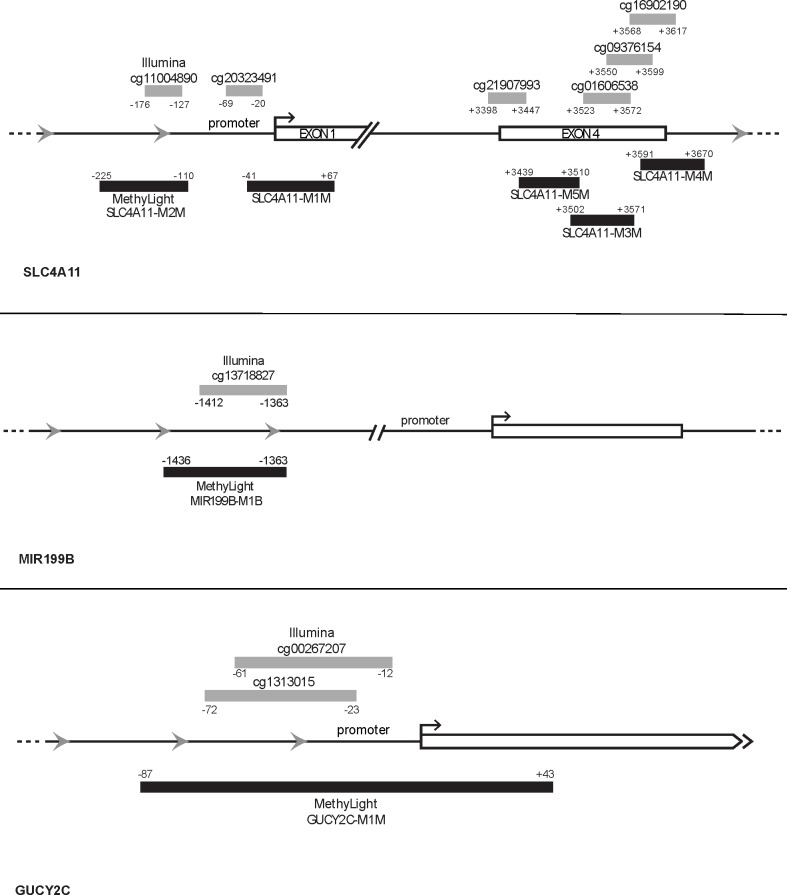
The location of Illumina Infinium HM450 array probes and MethyLight reactions for genes *SLC4A11* and *GUCY2C*.

To validate the array-based data using another DNA methylation detection assay, MethyLight analysis was performed on an additional cohort of FECD patient and control corneal samples (Table 3). MethyLight, a real-time-PCR based assay, [38], was used because of its ability to examine DNA methylation in FECD samples using small amounts of DNA, and because of its ability to provide quantitative estimates of DNA methylation levels [39]. A complete list of all MethyLight reactions is provided in S1 Table. MethyLight reactions were designed to represent the five *SLC4A11* probes (two promoter, three gene body), one *GUCY2C* probe (promoter), and one *MIR199B* probe (promoter) that were all found to be hypermethylated in FECD samples (Fig 5). Multiple CpG sites in *SLC4A11* were chosen for validation because the HM450 methylation array identified several significantly hypermethylated CpGs in the *SLC4A11* promoter and gene body regions in FECD CE tissues. Furthermore, a prior serial analysis of gene expression (SAGE) study found *SLC4A11* to be underexpressed in the CE of Fuchs patients [35]. *SLC4A11* was also of particular interest because *SLC4A11* mutations have been reported to be associated with some cases of late-onset FECD [40, 41]. *GUCY2C* was selected for validation because it had the most significantly methylated promoter CpG site in the FECD samples (Table 2). MicroRNA expression has been found to be decreased in FECD CE, with *MIR199B* being the most underexpressed by array analysis [42]. Our HM450 array analysis unveiled promoter DNA methylation of *MIR199B*.

**Table 3:** Demographics of study participants for MethyLight analysis.

	All FECD (n = 12)	Control (n = 3)	Analyzed FECD (n = 7)
**Age (SD)**	67.79 (11.01)	70.00 (6.2)	69 (11)
Age range	54.6–87.2	63–75	54.6–87.2
**Sex**			
Male	6 (50%)	1 (33%)	3 (43%)
Female	6 (50%)	2 (66%)	4 (57%)
**Study Eye**			
Right	4 (33%)	1 (33%)	2 (29%)
Left	8 (66%)	2 (66%)	5 (71%)
**Procedure**			
DSAEK	7 (58%)	0 (0%)	5 (71%)
DMEK	5 (42%)	3 (100%)	2 (29%)
**FECD Measures**			
Guttae	2.64	0	2.64
Pachymetry	639	N/A	623

**Fig 6:**
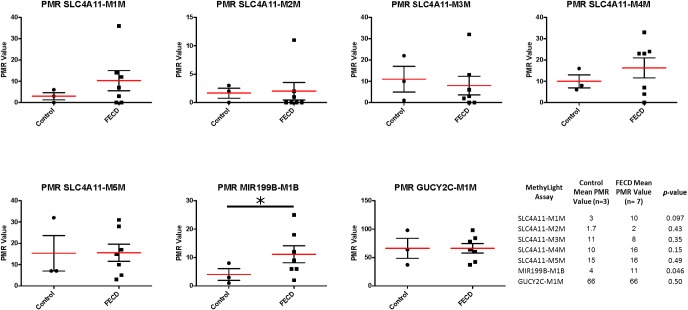
MethyLight analysis for *SLC4A11*, *MIR199B*, *and GUCY2C* in FECD and control endothelial tissues. Quantitative DNA methylation-sensitive real-time polymerase chain reaction (MethyLight) analyses performed on samples from three control samples and seven FECD samples. MethyLight data are presented as percent of methylated reference (PMR). The table lists the mean PMR values for the control and FECD samples and p-values for each MethyLight assay.

The MethyLight data confirmed DNA hypermethylation in the FECD samples compared with control samples (Fig 6). Of seven MethyLight assays tested, five assays (SLC4A11-MIM, SLC4A11-M2M, SLC4A11-M4M, SLC4A11-M5M, MIR199B-M1B) gave higher mean DNA methylation values (Percent of Methylated Reference, PMR) in the FECD samples compared with the control samples (Fig 6). A PMR cut-off of 10 was used to dichotomize methylated and unmethylated samples. Using this cut-off, two reactions (SLC4A11-MIM and MIR199B-M1B) were methylated in the FECD samples but were unmethylated in the control samples. For the SLC4A11-M1M reaction, the average PMR values were 10 and 3 for FECD and control samples, respectively. For the MIR199B-M1B reaction, the mean PMR value was 11 for FECD samples and 4 for control samples. In addition, the mean PMR value of the SLC4A11-M4M reaction was higher in the FECD samples (PMR = 16) compared with control samples (PMR = 10) (Fig 6). These results showed similar DNA methylation trends in the FECD and control samples using two independent sets of tissue samples and two different DNA methylation detection technologies.

## Discussion

Epigenetic mechanisms are not well studied within the field of ophthalmology, particularly with respect to corneal diseases. Epigenetic marks may be modified by environmental exposures [43] and consequently provide a mechanistic link between environmental risk factors and the etiology of diseases. It has been postulated that epigenetic changes might partially explain the late onset and progressive nature of some ocular diseases such as macular degeneration and glaucoma [44, 45], which are not fully explained by known mutations. The same may be true in late-onset FECD where the disease is manifest late in life and a single causative gene mutation has not yet been identified.

Our understanding of the genetic, epigenetic, and molecular mechanisms of Fuchs endothelial corneal dystrophy is still evolving. Some corneal dystrophies have been mapped to single genetic loci [46], including an early-onset form of FECD [47], but late-onset FECD has been linked to a variety of genetic and environmental factors [48]. This current study reports epigenetic changes, specifically alterations in DNA methylation, which occur in the corneal endothelial tissue of patients with late-onset FECD. Using a comprehensive, genome-scale DNA methylation array, we observed a significant difference in the DNA methylation profile of endothelial tissue of FECD patients compared with normal control patients. These changes are largely independent of age and sex-related effects. We found a large number of consistently differentially methylated probes between the FECD and control samples. Of the top 20 significantly differentially methylated probes, approximately half are annotated to genes that have a role in cytoskeletal organization. Gene ontology analysis further supported a potential role for DNA methylation changes in genes related to cytoskeletal organization and also identified aberrant DNA methylation patterns in genes involved in fluid and ion transport, hematopoetic cell differentiation, and metabolic processes as potentially involved in FECD. Our findings suggest that alterations in DNA methylation may contribute to FECD pathogenesis by modifying the barrier, metabolic, and fluid transport functions of the corneal endothelium.

Promoter DNA hypermethylation can be associated with transcriptional silencing of genes [49]. We observed significant promoter DNA hypermethylation in FECD CE of gene families involved in cellular metabolic processes. Previous studies have demonstrated a key role of oxidative stress in FECD pathogenesis [50]. Exposure of normal corneal endothelial cells to oxidative stress results in a loss of proliferative capacity and reduced cellular function [51]. Furthermore, increased expression of cellular senescence-related genes has been observed in tissue from FECD patients [52]. Intriguingly, our finding of promoter DNA hypermethylation of genes involved in cellular metabolism is consistent with a prior serial analysis of gene expression (SAGE) study that compared gene expression profiles of normal human CE with Fuchs’ CE [35]. This study showed that underexpressed transcripts exceed overexpressed genes in Fuchs’ CE, and that mitochondrial transcripts, in particular, are systematically depleted in FECD [35]. These results support the important role of energy metabolism in the pathophysiology of endothelial cellular dysfunction in FECD.

Our study also found promoter DNA hypermethylation of the membrane transporter gene *SLC4A11* in FECD patient samples by both HM450 array and MethyLight analyses. Down-regulated expression of *SLC4A11* in FECD patients has been demonstrated by prior SAGE analysis [35]. FECD is genetically heterogeneous and a small subset of individuals with late onset, dominantly-inherited FECD displayed *SLC4A11* mutations [31, 40]. *SLC4A11* mutations have also been associated with the autosomal recessive form of another corneal dystrophy, congenital hereditary endothelial dystrophy (CHED) [40, 53, 54]. *SLC4A11* is a member of the SLC4 family of bicarbonate transporters and is an integral membrane protein abundantly expressed by CECs [55–57]. SLC4A11 mediates Na^+^-independent and Na^+^-coupled H^+^ flux and Na^+^-coupled OH^−^ transport, but does not transport B(OH)_4_^−^BOH4− or HCO_3_^−^HCO3^−^ [57, 58]. In addition to ion transport, SLC4A11 has been shown to function in a water transport mode in the presence of an osmotic gradient [56]. The increased loss of corneal endothelial cells in patients with *SLC4A11* gene mutations has been proposed to be due to an increased propensity for these cells to undergo apoptosis [59].

Our Illumina HM450 DNA methylation array analysis also identified differentially methylated probes for a high number miRNAs. It is becoming increasingly clear that DNA methylation plays a central role in regulating miRNA expression [60–62]. Widespread miRNA downregulation has recently been observed in FECD [42]. Array analysis identified the down-regulation of 87 miRNAs in FECD compared with normal endothelium, with *MIR199B* having the largest change in expression [42]. We found *MIR199B* promoter DNA hypermethylation in FECD patient samples by both Illumina HM450 array and MethyLight analyses. Our results suggest miRNA promoter DNA hypermethylation as an important mechanism in their down-regulation in FECD.

Interestingly, we found that promoters of genes involved in cytoskeletal organization tend to be hypomethylated in FECD. Cytoskeletal factors, including actin filaments and microtubules tethered to tight and adherens junctions, are critical to the barrier integrity of the corneal endothelium and help to maintain corneal clarity by restricting fluid leakage into the corneal stroma [3]. Others have shown that modification of the cytoskeletal organization of the cornea can be effective in treating corneal endothelial dysfunction in FECD. The Rho/Rho-kinase (ROCK) pathway regulates the cytoskeleton, cell migration, cell proliferation, and apoptosis [63]. Inhibition of this pathway in CECs with a selective ROCK inhibitor result in the inhibition of apoptosis and the promotion of adhesion and proliferation [63]. These findings support the importance of cytoskeletal factors in normal corneal endothelial barrier function and repair, and as potential therapeutic targets for FECD.

The causal mechanism of gene body DNA methylation is not well understood, but DNA methylation in the transcribed regions of genes has been positively correlated with gene expression [64]. We found FECD-specific gene body DNA hypomethylation of gene families associated with ion and water transport. The pump functions of the corneal endothelium are important in maintaining corneal transparency. The above described SAGE analysis revealed a significant decrease in the bicarbonate transporter-related protein-1 (*BTR1*) in FECD [35]. The water transporting protein, aquaporin-1 (AQP1), that helps regulate corneal stromal hydration has also been shown to be underexpressed in FECD CE compared with normal CE [36]. More recently, expression of Na^+^/K^+^ ATPase and four isoforms of monocarboxylate transporters (MCTs) have similarly been found to be down-regulated in FECD CE [37]. Loss of ion transporters is a therefore a mechanism strongly linked to FECD, and our finding that gene bodies of ion transporters are less methylated and thus less often transcribed in FECD is consistent with these previously described changes in transport activity in FECD.

The limitations of this study include the inability to assay regions of the genome without annotated probes. The number of FECD patient and control samples analyzed was another limitation. Although the CE tissue of 15 FECD patients was collected, only 9 samples (60% of samples) had sufficient DNA extracted for array analysis. A minimum of 200 ng of genomic DNA is required for array analysis but for a large number of patients, especially those with more severe clinical findings associated FECD, even this amount of DNA was unobtainable due to the low number of healthy endothelial cells associated with their disease. Low DNA yield from the FECD samples was also an issue for the MethyLight validation studies. Because the FECD patients with more severe clinical findings could not be included in our analysis for this reason, it is possible that more significant DNA methylation differences may have been observed had these samples been included. The low amount of extractable DNA from the FECD samples precluded our ability to perform simultaneous methylation and expression analyses.

Taken together, we report the first genome-scale study of DNA methylation in FECD, and demonstrate that consistent DNA methylation alterations occur in the CE of FECD patients. These changes may contribute to the etiology and progression of FECD. In particular, we have identified significant DNA methylation changes in members of gene families associated with cytoskeletal organization, cellular metabolism, and ion transport, all pathways with known connections to FECD. During the last few decades, an increasing number of drugs targeting DNA methylation have been developed and have been used primarily in cancer therapies [65]. Unlike mutagenic events, epigenetic modifications are usually reversible. Our findings suggest altered DNA methylation may represent a new candidate therapeutic target in FECD. Further studies testing drugs that target global methylation may reveal a promising and novel approach to treating FECD.

## Materials and methods

### Ethical compliance

Institutional Review Board (IRB)/Ethics Committee approval was obtained from the University of California, San Francisco Human Research Protection Program (Study Number 11–07020). Written informed consent was obtained from all participants. Protected health information was masked according to HIPAA privacy standards and patient database was managed securely in Research Electronic Data Capture (REDCap) [66]. All of the described research adheres to the tenets of the Declaration of Helsinki.

### Subjects and selection criteria

Corneal endothelium was collected from FECD patients undergoing endothelial keratoplasty by a single surgeon (D.G.H.) at the University of California, San Francisco. Patients with a diagnosis of FECD and scheduled for endothelial keratoplasty with Dr. David Hwang between the dates of 2/12/2013 and 10/27/2014 (for Infinium Human Methylation450 BeadChip analysis) and 2/25/2015 and 12/9/2015 (for MethyLight analysis) were recruited for the study. The recruitment process included: (1) Review of the medical record by the principal investigator to identify participants with a clinical diagnosis of FECD; (2) Prior to the date of surgery, the principal investigator called the participant to inform them of the study and its risks and benefits; (3) On the date of surgery, a study coordinator met with the participant to review the study summary and written consent was obtained from consenting participants. Patient information (age, sex, affected eye, guttata score, pachymetry, past medical history) was collected with permission from electronic medical documentation of clinical examinations. Guttata score [67] and pachymetry [68] were taken from the most recent office visit prior to the procedure. Patients selected had no previous consumption of immunomodulatory medications and medications with known effects on epigenetic mechanisms. Non-FECD samples were obtained from an eye bank (SightLife, Seattle WA; and San Diego Eye Bank, San Diego CA). CE of these control samples was obtained from DMEK prepared central corneal tissue and processed in the same manner as the FECD samples. Patient and control cohorts were stratified by age and gender to minimize the effect of these factors on subsequent inferences of differential methylation patterns in FECD. Information about why the eye bank control samples were not suitable for transplantation and systemic co-morbidities of controls and FECD subjects are provided in S2 Table.

### DNA extraction and bisulfite conversion

CE was immediately immersed in PBS and RNAlater solution (Qiagen, Hilden, Germany) following surgical removal. DNA was extracted using Macherey Nagel NucleoSpin® Tissue XS according to manufacturer instructions (Macherey Nagel, GmbH & Co. KG, Germany). Briefly, tissue was pre-lysed with a proteinase K buffer at least 4 hours at 56°C and then incubated at 70°C for 5 minutes in lysis buffer. Binding conditions were adjusted with ethanol, and then lysate was loaded onto a silica column and washed twice. DNA was eluted into 20 μl of water and stored at -20°C. DNA bisulfite conversion was performed using the Zymo EZ DNA Methylation Kit (Zymo Research, Irvine, CA). A panel of MethyLight control reactions [69] measured bisulfite conversion completeness and the amount of available bisulfite-converted DNA. Samples without sufficient bisulfite-converted DNA were excluded from study. Infinium FFPE DNA Restoration was used to improve the quality of the resulting bisulfite-DNA to be used in HM450 data production (Illumina Inc., San Francisco, CA).

### DNA methylation microarray and statistical analysis

Following DNA extraction and bisulfite conversion, samples were hybridized to the Illumina Infinium Human Methylation450 BeadChip at the USC Norris Molecular Genomics Core Facility according to manufacturer protocols (Illumina Inc., San Francisco, CA). The IDAT files are available on the GEO DataSets database [accession number GSE94462; National Center for Biotechnology Information (NCBI), Bethesda, MD, USA]. All statistical analyses were executed in the R Anon. R: The R Project for Statistical Computing. Available at: http://www.r-project.org/ [Accessed June 18, 2015] programming environment using custom scripts available upon request from R.B.; elements of low-level array processing and quality control were performed using *methylumi* Anon. Bioconductor—methylumi. Available at: http://www.bioconductor.org/packages/release/bioc/html/methylumi.html [Accessed June 18, 2015] and *methAnalysis* Anon. Bioconductor—methyAnalysis. Available at: http://www.bioconductor.org/packages/release/bioc/html/methyAnalysis.html [Accessed June 18, 2015] packages. After excluding probes mapping to genomic regions containing known SNPs, raw intensity values were color corrected and then normalized using the smoothed quantile approach. We then calculated probe-level M values, which we locally smoothed (250bp windows) to stabilize DNA methylation estimates. The data derived from two arrays (hybridized with samples derived from a male and a female FECD donor) were rejected due to poor hybridization quality and were excluded from subsequent analyses. A summary of the samples collected and the samples eventually used for final analysis is shown in Table 1.

We identified differentially methylated probes using the following probe-wise linear model:

$$y_{ijkl}=a_{i}+\varsigma_{j}+\phi_{k}+\varsigma \phi_{jk}+\varepsilon_{ijkl}$$

In this framework, the normalized M-value of a probe derived from an array hybridized with DNA extracted from individual *l* of age *i*, sex *j*, and FECD disease state *k* is defined as a linear combination of fixed effects. Specifically, the model captures variation in DNA methylation levels related to the donor’s age (*α*, modeled as a continuous variable), sex (*ς*, fixed effect), and disease status (*ς*, fixed effect); we also included an interaction term, *ςϕ*, to enable detection of any changes in DNA methylation that are correlated with FECD in a sex-specific manner, and a residual term *ε* to capture unexplained variance, assumed to be Normally distributed with variance *σ*^*2*^. We determined the evidence for differential methylation with respect to each effect in the framework of the linear model using a Wald test, which we summarized as *P*-values. P-values were adjusted for multiple testing using the FDR approach of Benjamini & Hochberg, although we note that DNA methylation array probes are not strictly independent and these values likely imprecisely capture the true false positive rate.

All ontological category enrichment analyses were performed using GOseq [32] as described previously [33]. Briefly, we assigned a gene-level *Q*-value equal to the smallest *Q*-value observed among all of the probes on the array annotated to the corresponding gene, and then generated gene-wise weights calculated from the number of probes annotated to each gene. Genes with Q-values significant at an FDR of <0.01 were considered differentially expressed. When testing for ontological enrichment within subsets of the data (e.g., among promoter-annotated probes) we recalculated genewise *Q*-values on the basis of the corresponding probes and only considered qualifying genes (e.g., genes with probes annotated to the corresponding region) in the background set.

### MethyLight array design and statistical analysis

The Illumina HM450 results were screened to identify probes for validation. The following probes were chosen because they were significantly differentially methylated in FECD samples compared to the control samples: 5 probes from the SLC4A11 promoter and gene body (cg20323491, cg11004890, cg09376154, cg16902190, and cg21907993), 1 probe from the GUCY2C promoter (cg00267207), and 1 probe from the MIR199B promoter (cg13718827). Sequence details for these probes are provided in S1 Table.

DNA methylation levels were measured at the USC Norris Molecular Genomics Core Facility using MethyLight technology. MethyLight is a quantitative, TaqMan-based real-time PCR assay using bisulfite converted DNA as a substrate [39, 70]. In brief, genomic DNA (200ng– 500ng) for each sample was converted with bisulfite using the Zymo EZ DNA Methylation kit (Zymo Research, Irvine, CA) as specified by the manufacturer. A bisulfite-converted, M.*Sss*I-treated DNA sample was used as a methylated reference. MethyLight data were reported as a ratio between the value derived from the real-time PCR standard curve plotted as log(quantity) versus threshold C(t) value for each gene-specific methylation reaction and likewise for a methylation-independent control reaction based on interspersed *ALU* repeats [38, 69, 71]. This calculation was performed for both the sample and an M.*Sss*I-treated genomic DNA sample, which were used as a constant methylated reference. We calculated the percent of methylated reference (PMR) for each sample as: 100 X (GENE / ALU)_sample_ / (GENE / ALU)_M.*Sss*I-Reference_. We dichotomized all PMR values at 10. This threshold was chosen as a point sufficiently above background measurements and low rates of stochastic DNA methylation. We used this threshold previously for determining DNA methylation in colorectal cancer [71].

## Supporting information

S1 Table List of MethyLight primer and probe sequences. (XLSX)

S2 Table Clinical information about eye bank control samples and FECD patients. (XLSX)
